# Safety and Effectiveness of COVID-19 Vaccines: Results from a Cross-Sectional Survey among Staff, Workers and Students at an Egyptian University

**DOI:** 10.3390/vaccines10060846

**Published:** 2022-05-26

**Authors:** Marwa S. Hamza, Rajiv Tikamdas, Noha S. El Baghdady, Moustafa Sayed, Amani S. Elbarazi, Osama A. Badary, Mohamed M. Elmazar

**Affiliations:** 1 Clinical Pharmacy Practice Department, Faculty of Pharmacy, The British University in Egypt, El-Sherouk City, Cairo 11837, Egypt; rajiv.harish@bue.edu.eg (R.T.); noha.elbaghdady@bue.edu.eg (N.S.E.B.); helmy.mostafa@bue.edu.eg (M.S.); amani.safwat@bue.edu.eg (A.S.E.); osama.badary@bue.edu.eg (O.A.B.); 2 Pharmacology and Biochemistry Department, Faculty of Pharmacy, The British University in Egypt, El-Sherouk City, Cairo 11837, Egypt; mohey.elmazar@bue.edu.eg

**Keywords:** COVID-19 vaccine, SARS-CoV-2 vaccine, post-vaccination symptoms, side effects, Egypt

## Abstract

Mass vaccination is the most effective strategy against the spread of the COVID-19 pandemic. However, concerns about the vaccine’s safety and effectiveness remain a huge obstacle to vaccine acceptance. The aim of the present study was to explore different COVID-19 vaccine outcomes, including the development of adverse events and/or COVID-19 infection following COVID-19 vaccination. A cross-sectional study was conducted by distributing an online survey targeting staff and students at the British university in Egypt. A total of 637 participants fully completed the survey. Of these, 609 (95.6%) participants received the COVID-19 vaccine. Only 12.6% of the total vaccinated participants reported COVID-19 infection after vaccination. Of these, only 2.8% reported having severe symptoms while 9.9% reported having no or mild symptoms. The most common side effects reported after the first vs. second dose were headache (36.3% vs. 14.6%), tiredness and fatigue (26.9% vs. 10.7), and fever (25.6% vs. 6.7%). In conclusion, the present study explored different COVID-19 vaccine outcomes where the overall incidence of side effects is higher after the first dose than after the second dose. There is a relationship between COVID-19 vaccines’ side effects and gastrointestinal disorders, gender, and the type of COVID-19 vaccine. Post-vaccination symptoms were more frequently reported in women compared to men and more frequent with viral vector vaccines compared to other types. The effectiveness of different types of COVID-19 vaccines was confirmed by the lower incidence rate of post-vaccination COVID-19 infection.

## 1. Introduction

COVID-19 disease is one of the leading causes of death in the world [1]. Because this disease is contagious and has been anticipated to have a high fatality rate, disruptions and dangers to daily life are severe [2]. However, a number of preventive measures, including social distancing, quarantining, face-covering, ventilation of indoor spaces, hygienic practices, thorough screening tests, and execution of government legislation, have lessened the health impact [3]. The most reliable means to ensure control over this pandemic is to develop effective vaccines to control its spread [4]. Different types of COVID-19 vaccines have been approved and are being used all over the world, including those of Pfizer BioNTech, AstraZeneca, Moderna, Johnson & Johnson, Sinopharm, Sinovac, Sputnik V, and COVAXIN Vaccines [5,6]. Between December 2020 and January 2022, Egypt has received around 60 million vaccine doses from different countries within the national COVID-19 immunisation program. By the end of 2021, more than 55 million vaccine doses had been administered, assuming that each person requires two doses, which would be enough to vaccinate approximately 35.5% of the country’s population [7].

Due to the urgency of the pandemic, the quick development of COVID-19 vaccines and technological advancements have led to various rumours [8], which cause vaccine hesitancy. Also, it is believed that the population’s decision on refusing or delaying vaccination is mainly due to a lack of knowledge about the relative benefit-to-risk ratio [9,10,11].

Various post-vaccination side effects have been reported and believed to resolve within a few days, which include injection site pain or redness or swelling, fatigue, headache, chills, fever, muscle and joint pain, nausea, and swollen lymph nodes [9,10]. Vaccine reactions and side effects are distinct from one another in terms of physical symptoms. Redness, swelling, or discomfort at the injection site, as well as systemic symptoms such as fever or myalgia, can all be signs of reactogenicity, which is produced by an excessive inflammatory response to vaccination. In addition, females subjected to COVID-19 vaccines may experience menstrual abnormalities [12]. The occurrence of side effects is considered one of the main causes of vaccine hesitancy among people, which represents a huge obstacle to the success of vaccination strategies [13].

During the COVID-19 pandemic, mental distress may result in vaccination avoidance and refusal [14]. People who received vaccinations reported an improvement in social skills, which may be related to better mental health. However, a number of psychological variables (such as anxiety) are thought to play a role in the occurrence and severity of vaccine-related adverse effects [15]. The aim of the present study was to determine the short-term physical and psychological side effects of different COVID-19 vaccines reported by workers and students at the British University in Egypt, in addition to the incidence of post-vaccination COVID-19 infection.

## 2. Materials & Methods

### 2.1. Study Design and Setting

An anonymous online cross-sectional survey was designed on Survey Monkey and a link was sent to participants via institutional email. It was carried out from 1 January 2022 to 31 March 2022, among administrative staff, teaching staff, researchers, university employees, and students at the British University in Egypt.

### 2.2. Study Sample, Sampling, and Sample Size Calculation

The eligible study population comprised the entire university population (about 11,000 people), including administrative, teaching, and research staff, researchers, university employees, and students. The sample size was calculated by using the online sample size calculator RaoSoft*^®^*. Based on an estimated population of 11,000 workers and students and on an anticipated response of 50%, the minimum required sample size was 372 participants with a confidence level of 95% and a 5% margin of error.

### 2.3. Ethical Considerations

In accordance with the institution*’*s established standards, all ethical criteria were followed. Anonymity was ensured by not collecting any personal data (such as names or other identifiers). During the research process, all of the necessary ethical considerations were taken into account. All of the participants were fully informed of the study*’*s purpose and scope before they agreed to participate in the survey. A notification on the Survey Monkey platform informed the participants of the estimated completion time (5 min). To prevent anyone from taking the survey more than once, we recorded each respondent*’*s IP address, and no more than one response was allowed per IP address (ensured by the Survey Monkey response feature). Prior to participating, all participants were given assurances of complete anonymity and confidentiality. As a final reminder, all participants were informed that their participation in the survey was entirely voluntary.

### 2.4. Data Collection

Pre-validated questionnaires [16,17] were modified after a comprehensive literature search. The initial draft was sent to a group of experts, chosen according to their experience and expertise in related fields, to appraise the questions in terms of relativity, simplicity, and importance. A pilot study was conducted on 10 subjects, to test the questionnaire’s validity. Following a group discussion, the questionnaire was completed. The data from the pilot study was removed from the final analysis. The questionnaire was composed of two main categories. The first section covered the background data such as gender, level of education, age, and any previously reported COVID-19 infection. The second part of the questionnaire was designed to collect information on data related to COVID-19 vaccination status. Questions were targeted at identifying the type of vaccine received, the number of doses, side effects, and onset time (i.e., after the first or second dose). A list of side effects was provided to participants to choose from, which included flu-like symptoms, pain at the site of injection, dyspnoea, headache, and tachycardia, in addition to mentioning if they experienced other side effects not listed. Precautionary actions were taken to protect privacy and freedom of terminating the survey at any time during the study. In the pilot sample, the knowledge questionnaire*’*s Cronbach’s alpha coefficient was 0.84, indicating very reliable internal consistency [18].

Moreover, participants were asked about their self-reported mental suffering after vaccination, as indicated by the four-item Patient Health Questionnaire (PHQ-4). The four-item Patient Health Questionnaire is used to assess mental distress (PHQ-4) [19]. Two questions assess depressive symptoms, while the other two assess anxious symptoms. Each item is rated on a scale of 0 to 4, and the responses are added up to form an index ranging from 0 to 16, with higher values indicating greater degrees of mental discomfort. Earlier research has established the PHQ-4*′*s validity and reliability [20].

### 2.5. Statistical Analysis

All statistical tests were executed by the Statistical Package for the Social Sciences (SPSS) version 23.0 (SPSS Inc., Chicago, IL, USA, 2020). The normality of data was evaluated using the Shapiro–Wilk test with a significance level *p*-value < 0.05. In the pilot study, Cronbach’s alpha coefficient test was used to measure internal consistency. Primarily, descriptive statistics were used to present and summarise the categorical variables like gender, age categories, profession, work experience, region, medical anamneses, COVID-19-related anamneses, and individual side effects by frequency (*n*) and percentage (%). A Chi-squared test (χ^2^) was used to estimate the association between different demographic and medical characteristics and different types of COVID-19 vaccines.

Binary logistic regression analysis was carried out to find the odds ratio for the occurrence of post-vaccination symptoms. All inferential tests were carried out assuming a confidence level (CI) of 95% and a *p*-value of < 0.05.

## 3. Results

### 3.1. Demographic and Clinical Characteristics of the Participants

A total of 637 participants fully completed the survey. Of these, 609 (95.6%) participants received the COVID-19 vaccine, which indicates a high vaccination rate. The most common reasons for receiving the vaccine were self and others’ protection and vaccination being required for work/university/other activities. On the other hand, the most commonly reported reasons for not receiving the vaccine were having concerns about vaccine safety, having recently recovered from COVID-19, and difficulty scheduling or getting an appointment. More than half of the non-vaccinated participants, however, reported that they planned to receive the vaccine.

Different types of vaccines were received by the vaccinated participants where 307 (50.4%) participants received inactivated vaccine (37.8% Sinovac and 12.6% Sinopharm), 240 (39.4%) participants received viral vector vaccine (27.6% AstraZeneca/Oxford, 10.8% Johnson & Johnson and 1% Sputnik V), and 62 (10.2%) participants received mRNA vaccine (9% Pfizer-BioNTech and 1.1% Moderna). A total of 609 vaccinated participants were included in the downstream analyses where 395 (64.9%) were females and 214 (35.1%) were males; 447 (73.4%) were aged between 18 to 29 years and 140 (22.9%) were aged between 30 to 59 years; the educational level of 365 (59.9%) was undergraduate, 88 (14.4%) had a bachelor’s degree and 156 (25.6%) had a postgraduate degree; 389 (63.9%) were students, 151 (24.8%) were academic staff and 69 (11.3%) were administrative and support staff; 562 (92.3%) were residents of Cairo and 47 (7.7%) were from other governorates, 109 (17.9%) were smokers, 132 (21.7%) received a flu shot this year, 68 (11.2%) reported suffering from an allergy to some types of foods or medicines, 266 (43.7%) reported at least one non-communicable disease, and 114 (18.7%) reported taking medications. There was a significant association between the type of vaccine received and the participants*’* age (*p*-value < 0.001), educational level (*p*-value < 0.001), employment status (*p*-value < 0.001), and place of residence (*p*-value = 0.01) (Table 1).

### 3.2. COVID-19-Related Anamneses

The majority of vaccinated participants (82.1%) received two doses. In addition, 2.1% of the participants received more than two doses (booster doses). Two hundred and fourteen participants (35.1%) reported having a previous COVID-19 infection before vaccination, compared to only 77 (12.6%) who reported having COVID-19 infection after vaccination. Of those who reported post-vaccination COVID-19 infection, only 17 (2.8%) reported having severe symptoms while 60 (9.9%) reported having no or mild symptoms, suggesting significant vaccine effectiveness. Regarding symptoms following vaccination, 379 participants (62.2%) reported having symptoms, with the majority being mild to moderate symptoms. Almost half of the participants reported an onset of symptoms within 12 h and a duration ranging from less than a day up to 3 days. The majority of the participants’ responses to relieving the vaccination symptoms were resting at home and taking painkillers. There were significant associations between the type of vaccine received and the number of vaccine doses received (*p*-value < 0.001), occurrence of symptoms (*p*-value < 0.001), onset of symptoms (*p*-value < 0.001), duration of symptoms (*p*-value = 0.046) as well as response to post-vaccination symptoms (*p*-value < 0.001) (Table 2).

### 3.3. Safety and Effectiveness of COVID-19 Vaccines

#### 3.3.1. Reported COVID-19 Vaccines Side Effects after First and Second Dose

**Figure 1:**
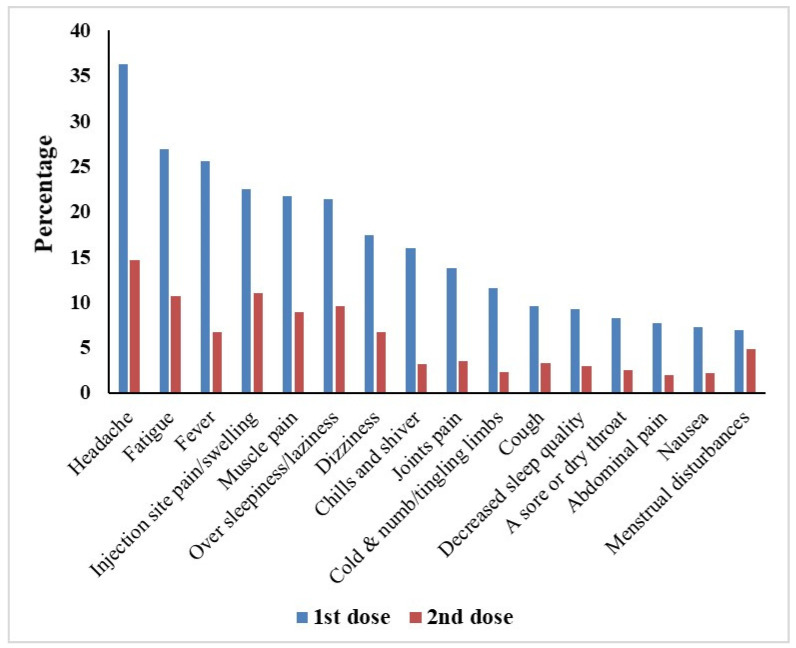
Most common COVID-19 vaccine side effects reported by the participants after the first and second vaccine dose (*n* = 609).

The most common side effects reported after the first and second dose were headache (36.3% vs. 14.6%), tiredness and fatigue (26.9% vs. 10.7%), fever (25.6% vs. 6.7%), pain or swelling at the injection site (22.5% vs. 11%), muscle pain (21.7% vs. 8.9%), excessive sleepiness/laziness (21.3% vs. 9.5%), and dizziness (17.4% vs. 6.7%), which indicates lower incidence rates of side effects following the second dose compared to the first dose (Figure 1). Interestingly, menstrual problems in females were reported in 42 (10.6% of the female participants) and 29 (7.3% of the female participants) after the first and second dose, respectively. In addition, a case of male impotence following a Johnson & Johnson single dose was reported. Also, three cases of thrombocytopenia (two with AstraZeneca/Oxford vaccine and one with Sinovac vaccine) and one case of thrombosis with AstraZeneca/Oxford vaccine were reported after the second dose. Regarding side effects following the first dose, there was a significant association between the type of vaccine received and the following side effects: sore or dry throat (*p*-value = 0.003), chills and shiver (*p*-value < 0.001), clogged nose (*p*-value = 0.014), cold, numbness and tingling in limbs (*p*-value < 0.001), cough (*p*-value = 0.001), decreased sleep quality (*p*-value < 0.001), diarrhoea (*p*-value = 0.003), faster or irregular heartbeats (*p*-value = 0.014), runny nose (*p*-value < 0.001), dizziness (*p*-value < 0.001), fever (*p*-value < 0.001), haziness or lack-of-clarity in the eyesight (*p*-value = 0.001), headache (*p*-value < 0.001), joints pain (*p*-value < 0.001), muscle pain (*p*-value < 0.001), nausea (*p*-value < 0.001), over sleepiness or laziness (*p*-value < 0.001), pain or swelling at the injection site (*p*-value < 0.001), tiredness and fatigue (*p*-value < 0.001) as well as body sweating for no reason (*p*-value < 0.001). All of these side effects were most common with viral vector vaccines, especially the AstraZeneca/Oxford vaccine.

On the other hand, regarding side effects following the second dose, there was a significant association between the type of vaccine received and diarrhoea (*p*-value = 0.002), dizziness (*p*-value = 0.021), fever (*p* -value = 0.005), headache (*p*-value < 0.001), muscle pain (*p*-value < 0.001), over sleepiness or laziness (*p*-value = 0.025), pain or swelling at the injection site (*p*-value < 0.001), tiredness and fatigue (*p*-value = 0.027), vomiting (*p*-value = 0.026) as well as menstrual disturbance in females (*p*-value = 0.039). The detailed incidence rates of the various COVID-19 vaccine-associated side effects stratified by the type of COVID-19 vaccine received are shown in (Table 3 and Table 4).

Moreover, the present study reported that the occurrence of side effects was higher after the first dose than after the second dose. The mean of the ratio between the occurrence of side effects between the first dose and the second dose for all the side effects was 3.3 ± 0.2 (95% confidence interval [CI], 2.8 to 3.7). Interestingly, it was observed that the ratio of the occurrence of menstrual disturbance, particularly as a side effect, was 1.4 between the first and second dose, which differs significantly from the ratio between all the other side effects.

#### 3.3.2. Factors Associated with Post-Vaccination Symptoms and COVID-19 Infection

The association of post-vaccination symptoms and COVID-19 infection with various demographic and clinical characteristics was assessed as shown in Table 5. Regarding post-vaccination symptoms, there was a significant association between the development of symptoms and gender (*p*-value < 0.001), number of vaccine doses (*p*-value = 0.011), and type of vaccine received (*p*-value < 0.001) where symptoms were more likely to occur in females, those who received a single dose of the vaccine, and those who received viral vector vaccines, especially the AstraZeneca/Oxford vaccine. Regarding post-vaccination COVID-19 infection, there was a significant association between COVID-19 infection after vaccination and previous COVID-19 infection before vaccination (*p*-value = 0.005) where COVID-19 infection after vaccination was more likely to occur in those who had previous COVID-19 infection before vaccination. Interestingly, the incidence of COVID-19 infection after vaccination was significantly lower and there was no significant difference among vaccine types in terms of post-vaccination COVID-19 infection, suggesting a similar level of protection against COVID-19 infection.

#### 3.3.3. Risk Factors for Occurrence of Symptoms after of Vaccination

A binary logistic regression analysis was performed where the occurrence of symptoms after vaccination was used as the dependent variable and different patients’ characteristics as prognostic variables. There was a significant association between the occurrence of symptoms after vaccination and gastrointestinal disorders, gender, and type of vaccine. The risk of the occurrence of symptoms after vaccination increased by ~4.5 times in patients suffering from gastrointestinal disorders (95% confidence interval [CI], 1.274 to 16.218). For males, the occurrence of symptoms after vaccination decreased by 0.3 times more than for females (95% confidence interval [CI], 0.237 to 0.506). Moreover, for participants who received Johnson & Johnson vaccine and AstraZeneca, the occurrence of post-vaccination symptoms increased by 5.9 and 5.1 times more than for participants who received Sinovac (95% confidence interval [CI], 2.852 to 12.473; 3.159 to 8.376 respectively) (Table 6).

### 3.4. Mental Health Symptoms and Association with COVID-19 Vaccination

The PHQ-4 was used to determine whether or not participants had suffered from mental discomfort. As shown in Table 7, there is no statistical variation in the mental health status across COVID-19 vaccine types (*p*-value > 0.05) and the mean of the subjects’ PHQ-4 scores was 2.1 ± 3.1. Approximately 16.1% of the sample fulfilled the PHQ-4 criteria for mild mental distress, 9.2% for moderate mental distress, and 6% for severe mental distress. Additionally, and as shown by the PHQ-4 subscales, the mean of the individuals’ anxiety ratings was 0.8 ± 1.5 and the mean of the depression score was 1.2 ± 1.7. A score of three or above on each subscale of anxiety and depression is deemed positive for screening purposes; however, the individuals’ scores were within the normal range, suggesting neither anxiety nor depression. PHQ-4 scores did not differ significantly by the type of vaccine.

## 4. Discussion

### 4.1. Principal Findings and Previous Studies

In the present study, our research group studied the short-term side effects following the administration of different types of COVID-19 vaccines administered in Egypt. A total of 637 participants from the British University in Egypt fully completed the survey. Of these, 609 (95.6%) participants received COVID-19 and 28 participants did not receive the vaccine due to negative safety thoughts. More than half of the non-vaccinated participants, however, reported that they plan to receive the vaccine, which further indicates the increasing willingness to receive vaccination. Females represented a total of 67.9% of the unvaccinated participants. In a study that investigated COVID-19 vaccine hesitancy and related fears and anxiety, males reported higher willingness than females on average, but the gender difference was not significant [21].

It was obvious in our study that the overall incidence of side effects is higher after the first dose than after the second dose. Headache (36.3% vs. 14.6%), tiredness and fatigue (26.9% vs. 10.7), fever (25.6% vs. 6.7%), pain or swelling at the injection site (22.5% vs. 11%), muscle pain (21.7% vs. 8.9%), over sleepiness/laziness (21.3% vs. 9.5%), and dizziness (17.4% vs. 6.7%) were the most common side effects reported after the first vs. second dose. That was in harmony with a previous study that confirmed that the side effects were most common after the first dose [22]. The occurrence of side effects after the first dose was almost three times higher than after the second dose. That was confirmed by a previous study that reported that the side-effect severity was greater after the first dose of Sinopharm and AstraZeneca than after the second dose, but in contrast, the side-effect severity was greater after the second dose of the Pfizer vaccine than after the first dose [23]. On the other hand, another study showed a significant increase in the number of subjects who were suffering side effects after receiving the second dose of the vaccine compared to those who reported side effects after the first dose [24]. 

Speaking precisely, menstrual problems in females were reported in 42 participants (10.6% of the female participants) and 29 participants (7.3% of the female participants) after the first and second dose, respectively. This finding is similar to previous studies that showed that approximately 5% of females of reproductive age reported menstrual abnormalities [25]. Menstrual abnormalities were reported to be short-term and ranged from cycle and menstrual length changes to differences in menstrual associated symptoms, unscheduled bleeding, and changes in the quality and quantity of menstrual bleeding [26]. This may be attributed to immune activation and the inflammatory response [27,28]. Also, it was observed that the occurrence of menstrual disturbance was higher after the first dose than the second dose by 1.4 times. Although a previous study confirmed that the occurrence of menstrual irregularities was higher after the second dose, those abnormalities were found to self-resolve within two months [29]. Moreover, the present study reported that the ratio between the occurrence of menstrual disturbance after the first and second dose is significantly lower than the ratio between all the other side effects after the first and second dose.

With respect to the vaccine type related side effects, we reported a significant association between the type of the vaccine and some of the side effects after the first dose, such as abdominal pain, chills, decreased sleep quality, faster or irregular heartbeats, fever, haziness or lack-of-clarity in your eyes, headache, joint pain, muscle pain, nausea, over sleepiness or laziness, pain or swelling at the injection site, tiredness and fatigue, and body sweating. The highest side effect incidence was reported after the AstraZeneca vaccine. Similar findings were reported in a prospective observational study conducted in the UK. Adverse events were considerably greater in people who received one dosage of the AstraZeneca vaccine when comparing systemic effects following one dose of each vaccination [30].

Following the second dose, three female cases of thrombocytopenia (two with AstraZeneca/Oxford vaccine and one with Sinovac vaccine) and one case of thrombosis with AstraZeneca/Oxford vaccine were reported. These findings are highly consistent with the results reported in phase III clinical trials and vaccine fact sheets, and they are mostly reported for those who received the second dose [31,32]. Also, there was a significant association between the type of vaccine received and headache as well as muscle pain after the second dose administration, which were more common with Moderna and Sputnik V vaccines, respectively. Similar findings were reported by others who found that mRNA vaccines have a higher incidence of second dose side effects (Moderna vs. Pfizer) [33].

The majority of the side effects were mild to moderate and usually resolved within a few days of vaccination, as reported in many trials [24,31,32,33]. In the current study, most of the participants reported mild to moderate symptoms, which is concomitant with the results of Elgendy et al.*’*s study, as most of the participants felt mild or no symptoms after vaccination [23]. Almost half of the participants indicated that their symptoms began within 12 h and lasted from less than a day to three days. Also, most of the participants in the previously mentioned Egyptian study reported that the side effects appeared on the first day of the Sinopharm, AstraZeneca, and Pfizer vaccines and endured no more than three days [23].

There was an association between post-vaccination symptoms and the type of vaccine. Therefore, a binary logistic regression analysis was carried out to find out the risk factors for these post-vaccination symptoms. Being female, suffering from gastrointestinal diseases, and receiving either AstraZeneca or Johnson & Johnson increases the risk of the occurrence of post-vaccination symptoms. Furthermore, as shown in our results, there was a significant association between the different side effects such as abdominal pain, chills and shivers, decreased sleep quality, faster or irregular heartbeats, dyspnoea, fever, haziness or lack-of-clarity in your eyes, headache, joint pain, muscle pain, nausea, oversleeping or laziness, pain or swelling at the injection site, tiredness and fatigue, and body sweating for no reason and receiving the viral vector vaccines, especially the AstraZeneca/Oxford vaccine. Interestingly, previous studies reported that the viral vector-based vaccine was associated with more frequent systemic side effects [5,6,34].

There was a significant association between post-vaccination symptom development and gender. Around seventy-three percent of the participants who had post-vaccination symptoms were females, whereas 27.2% were males. That could explain the high hesitancy of females in receiving the vaccine. Regardless of whether vaccinated women had much greater rates of adverse effects, it was still very low for both genders. In the limited trials that have been conducted, the reduced propensity to immunise females did not appear to translate into lower vaccination rates [35,36]. Moreover, there was a significant association between gender, the number of vaccine doses, and the type of vaccine received, with symptoms occurring more frequently in females, those who received a single dose of the vaccine, and those who received viral vector vaccines, particularly the AstraZeneca/Oxford vaccine. Conspicuously, the thrombosis and thrombocytopenia side effects were reported with the AstraZeneca vaccine. Yet, there was no causal relationship discovered to relate incidents of thrombotic adverse effects to the AstraZeneca vaccination [37]. A previous COVID 19 infection before vaccination was significantly associated with post-COVID-19 infection, as infection was more likely to occur in those who had previous COVID-19 infection before vaccination. This could be explained by the fact that these participants were more vulnerable to infection. Controversially, a previous study reported that the naturally infected populations were less likely to be re-infected by COVID-19 than infection-free and vaccinated individuals. Although re-infected people did not develop severe disease, a significant percentage of naturally infected or vaccinated people were re-infected by the emerging variants [38]. More research is required to address this issue.

In order to study the effectiveness of COVID-19 vaccines in terms of post-vaccination infection in the current study, there was no significant difference among vaccine types in terms of post-vaccination COVID-19 infection, implying a similar level of protection against COVID-19 infection. A previous study reported that COVID-19 inactivated vaccines, adenovirus-vectored vaccines, and mRNA vaccines’ have 60%, 65%, and 90% efficacy respectively against asymptomatic, symptomatic COVID-19 infection, COVID-19 hospitalization, severe or critical hospitalization, as well as mortality [39]. Meanwhile, this outcome needs more studies to be confirmed. More research is needed to understand the vaccination profiles for immunocompromised patients, organ transplant recipients, and other comorbidities [40].

Because the COVID-19 vaccination influences mental health both directly and indirectly [41], we investigated the effect of the COVID-19 vaccine on mental health among the study participants. We claim that vaccination type is not related to the level of mental distress experienced by patients. Aside from that, many participants were afraid of becoming infected and subsequent death during the pandemic. In addition, feelings of security and decreased mortality were a result of COVID-19 vaccination for those who were vaccinated. Our findings are consistent with a previous study which reported that individuals experienced low levels of discomfort following their initial dose of the vaccine [42].

### 4.2. Strengths and Limitations

The current study’s diversity in participants’ age groups, educational backgrounds, and clinical characteristics allowed the assessment of the association between different sociodemographic characteristics and safety as well as the effectiveness of different types of COVID-19 vaccines. Also, the comparison of side effects of seven COVID-19 vaccines of different types including inactivated, viral vector, and mRNA vaccines adds significant value to the present study. Furthermore, differences in mental health status across types of COVID-19 vaccines were studied in the Egyptian population for the first time. The main limitations of the present study are related to self-reported data and information about COVID-19 vaccine side effects. In addition, the present study monitored short-term post-vaccination side effects; therefore, future studies of longer periods and a larger number of participants need to be carried out to assess the long-term side effects of different COVID-19 vaccines and to assess the post-marketing surveillance of COVID-19 vaccines.

## 5. Conclusions

The present study explored different COVID-19 vaccine outcomes, including the development of adverse events and/or COVID-19 infection following COVID-19 vaccination. The overall incidence of side effects is higher after the first dose than after the second dose. Headache, tiredness and fatigue, fever, pain or swelling at the injection site, muscle pain, excessive sleepiness or laziness, and dizziness, as well as the occurrence of menstrual problems in females after COVID-19 vaccination, were the most common side effects reported after the first and second doses. COVID-19 vaccination is not related to the level of mental distress among the participants. Moreover, the present study reported the relationship between COVID-19 vaccines’ side effects and gastrointestinal disorders, gender, and the type of COVID-19 vaccine. In addition, it reported that the post-vaccination symptoms are more frequently reported in women compared to men and more frequent with viral vector vaccines compared to other types. The present study revealed the effectiveness of different vaccine types administered in Egypt based on the significantly low post-vaccination COVID-19 infection reported. We believe that our data could be of value to helping governmental organisations in Egypt with the likelihood of different post-vaccination side effects on the basis of age, sex, occupation, chronic diseases, and the type of vaccine being administered.

## Figures and Tables

**Table 1:** Demographic and clinical characteristics of the participants according to type of vaccine (*n* = 609).

	Inactivated Vaccine		Viral Vector Vaccine			mRNA Vaccine		Total (*n* = 609) N, %	*p*-Value
	Sinovac (*n* = 230) N, %	Sinopharm (*n* = 77) N, %	AstraZeneca/Oxford (*n* = 168) N, %	Johnson & Johnson (*n* = 66) N, %	Sputnik V (*n* = 6) N, %	Pfizer-BioNTech (*n* = 55) N, %	Moderna (*n* = 7) N, %		
**Gender**									0.528
Female	144 (62.6%)	53 (68.8%)	102 (60.7%)	48 (72.7%)	4 (66.7%)	39 (70.9%)	5 (71.4%)	395 (64.9%)	
Male	86 (37.4%)	24 (31.2%)	66 (39.3%)	18 (27.3%)	2 (33.3%)	16 (29.1%)	2 (28.6%)	214 (35.1%)	
**Age**									<0.001
Under 18	0 (0.0%)	1 (1.3%)	0 (0.0%)	0 (0.0%)	0 (0.0%)	13 (23.6%)	0 (0.0%)	14 (2.3%)	
18–29	187 (81.3%)	59 (76.6%)	101 (60.1%)	51 (77.3%)	6 (100.0%)	37 (67.3%)	6 (85.7%)	447 (73.4%)	
30–39	27 (11.7%)	4 (5.2%)	33 (19.6%)	7 (10.6%)	0 (0.0%)	3 (5.5%)	0 (0.0%)	74 (12.2%)	
40–49	10 (4.3%)	10 (13.0%)	22 (13.1%)	5 (7.6%)	0 (0.0%)	0 (0.0%)	0 (0.0%)	47 (7.7%)	
50–59	5 (2.2%)	2 (2.6%)	10 (6.0%)	2 (3.0%)	0 (0.0%)	0 (0.0%)	0 (0.0%)	19 (3.1%)	
60–69	1 (0.4%)	0 (0.0%)	2 (1.2%)	0 (0.0%)	0 (0.0%)	0 (0.0%)	1 (14.3%)	4 (0.7%)	
More than 69	0 (0.0%)	1 (1.3%)	0 (0.0%)	1 (1.5%)	0 (0.0%)	2 (3.6%)	0 (0.0%)	4 (0.7%)	
**Educational Level**									<0.001
Undergraduate student	158 (68.7%)	49 (63.6%)	71 (42.3%)	36 (54.5%)	4 (66.7%)	41 (74.5%)	6 (85.7%)	365 (59.9%)	
Bachelor’s degree	39 (17.0%)	10 (13.0%)	27 (16.1%)	6 (9.1%)	1 (16.7%)	5 (9.1%)	0 (0.0%)	88 (14.4%)	
Postgraduate studies	33 (14.3%)	18 (23.4%)	70 (41.7%)	24 (36.4%)	1 (16.7%)	9 (16.4%)	1 (14.3%)	156 (25.6%)	
**Employment status**									<0.001
Student	166 (72.2%)	50 (64.9%)	78 (46.4%)	40 (60.6%)	4 (66.7%)	45 (81.8%)	6 (85.7%)	389 (63.9%)	
Academic staff	31 (13.5%)	18 (23.4%)	68 (40.5%)	26 (39.4%)	1 (16.7%)	7 (12.7%)	0 (0.0%)	151 (24.8%)	
Administrative staff	33 (14.3%)	9 (11.7%)	20 (11.9%)	0 (0.0%)	0 (0.0%)	1 (1.8%)	1 (14.3%)	64 (10.5%)	
Support staff	0 (0.0%)	0 (0.0%)	2 (1.2%)	0 (0.0%)	1 (16.7%)	2 (3.6%)	0 (0.0%)	5 (0.8%)	
**Place of residence**									0.01
Cairo	218 (94.8%)	71 (92.2%)	148 (88.1%)	64 (97.0%)	3 (50.0%)	51 (92.7%)	7 (100.0%)	562 (92.3%)	
Other governorates	12 (5.2%)	6 (7.8%)	20 (11.9%)	2 (3.0%)	3 (50.0%)	4 (7.3%)	0 (0.0%)	47 (7.7%)	
**Smoker**									0.45
Yes	47 (20.4%)	13 (16.9%)	26 (15.5%)	11 (16.7%)	0 (0.0%)	9 (16.4%)	3 (42.9%)	109 (17.9%)	
No	183 (79.6%)	64 (83.1%)	142 (84.5%)	55 (83.3%)	6 (100.0%)	46 (83.6%)	4 (57.1%)	500 (82.1%)	
**Received flu shot this year**									0.71
Yes	46 (20.0%)	19 (24.7%)	41 (24.4%)	16 (24.2%)	1 (16.7%)	8 (14.5%)	1 (14.3%)	132 (21.7%)	
No	184 (80.0%)	58 (75.3%)	127 (75.6%)	50 (75.8%)	5 (83.3%)	47 (85.5%)	6 (85.7%)	477 (78.3%)	
**Previous Allergies**									0.55
Yes	23 (10.0%)	13 (16.9%)	17 (10.1%)	8 (12.1%)	0 (0.0%)	7 (12.7%)	0 (0.0%)	68 (11.2%)	
No	207 (90.0%)	64 (83.1%)	151 (89.9%)	58 (87.9%)	6 (100.0%)	48 (87.3%)	7 (100.0%)	541 (88.8%)	
**Comorbidities**									0.34
Yes	93 (40.4%)	37 (48.1%)	82 (48.8%)	27 (40.9%)	2 (33.3%)	20 (36.4%)	5 (71.4%)	266 (43.7%)	
No	137 (59.6%)	40 (51.9%)	86 (51.2%)	39 (59.1%)	4 (66.7%)	35 (63.6%)	2 (28.6%)	343 (56.3%)	
**Medications**									0.067
Yes	31 (13.5%)	22 (28.6%)	36 (21.4%)	14 (21.2%)	1 (16.7%)	10 (18.2%)	0 (0.0%)	114 (18.7%)	
No	199 (86.5%)	55 (71.4%)	132 (78.6%)	52 (78.8%)	5 (83.3%)	45 (81.8%)	7 (100.0%)	495 (81.3%)	

Chi-squared test was used with a *p*-value ˂ 0.05.

**Table 2:** COVID-19-related anamneses of the participants according to type of vaccine (*n* = 609).

	Inactivated Vaccine		Viral Vector Vaccine			mRNA Vaccine		Total (*n* = 609) N, %	*p*-Value
	Sinovac (*n* = 230) N, %	Sinopharm (*n* = 77) N, %	AstraZeneca/Oxford (*n* = 168) N, %	Johnson & Johnson (*n* = 66) N, %	Sputnik V (*n* = 6) N, %	Pfizer-BioNTech (*n* = 55) N, %	Moderna (*n* = 7) N, %		
**No. of vaccine doses**									<0.001
Single dose	17 (7.4%)	2 (2.6%)	5 (3.0%)	63 (95.5%)	1 (16.7%)	8 (14.5%)	0 (0.0%)	96 (15.8%)	
Two doses	213 (92.6%)	72 (93.5%)	159 (94.6%)	2 (3.0%)	5 (83.3%)	44 (80.0%)	5 (71.4%)	500 (82.1%)	
More than two doses	0 (0.0%)	3 (3.9%)	4 (2.4%)	1 (1.5%)	0 (0.0%)	3 (5.5%)	2 (28.6%)	13 (2.1%)	
**Previous COVID-19 infection**									0.762
Yes	81 (35.2%)	30 (39.0%)	58 (34.5%)	24 (36.4%)	3 (50.0%)	17 (30.9%)	1 (14.3%)	214 (35.1%)	
No	149 (64.8%)	47 (61.0%)	110 (65.5%)	42 (63.6%)	3 (50.0%)	38 (69.1%)	6 (85.7%)	395 (64.9%)	
**Postvaccination COVID-19 infection**									0.64
No	201 (87.4%)	67 (87.0%)	149 (88.7%)	54 (81.8%)	4 (66.7%)	50 (90.9%)	7 (100.0%)	532 (87.4%)	
Yes with no symptoms	2 (0.9%)	1 (1.3%)	2 (1.2%)	3 (4.5%)	0 (0.0%)	1 (1.8%)	0 (0.0%)	9 (1.5%)	
Yes with mild symptoms	18 (7.8%)	7 (9.1%)	14 (8.3%)	8 (12.1%)	1 (16.7%)	3 (5.5%)	0 (0.0%)	51 (8.4%)	
Yes with severe symptoms	9 (3.9%)	2 (2.6%)	3 (1.8%)	1 (1.5%)	1 (16.7%)	1 (1.8%)	0 (0.0%)	17 (2.8%)	
**Postvaccination symptoms**									<0.001
No symptoms at all	118 (51.3%)	44 (57.1%)	31 (18.5%)	10 (15.2%)	1 (16.7%)	24 (43.6%)	2 (28.6%)	230 (37.8%)	
Yes, mild symptoms	84 (36.5%)	23 (29.9%)	53 (31.5%)	24 (36.4%)	2 (33.3%)	21 (38.2%)	4 (57.1%)	211 (34.6%)	
Yes, moderate symptoms	24 (10.4%)	9 (11.7%)	54 (32.1%)	18 (27.3%)	3 (50.0%)	8 (14.5%)	1 (14.3%)	117 (19.2%)	
Yes, severe symptoms	4 (1.7%)	1 (1.3%)	30 (17.9%)	14 (21.2%)	0 (0.0%)	2 (3.6%)	0 (0.0%)	51 (8.4%)	
**Onset of symptoms**									<0.001
Up to 4 h	45 (19.6%)	10 (13.0%)	13 (7.7%)	10 (15.2%)	1 (16.7%)	10 (18.2%)	0 (0.0%)	89 (14.6%)	
5 to 8 h	39 (17.0%)	7 (9.1%)	53 (31.5%)	19 (28.8%)	1 (16.7%)	6 (10.9%)	2 (28.6%)	127 (20.9%)	
9 to 12 h	12 (5.2%)	7 (9.1%)	38 (22.6%)	19 (28.8%)	2 (33.3%)	8 (14.5%)	3 (42.9%)	89 (14.6%)	
13 to 16 h	0 (0.0%)	1 (1.3%)	13 (7.7%)	3 (4.5%)	1 (16.7%)	3 (5.5%)	0 (0.0%)	21 (3.4%)	
17 to 20 h	3 (1.3%)	0 (0.0%)	4 (2.4%)	1 (1.5%)	0 (0.0%)	1 (1.8%)	0 (0.0%)	9 (1.5%)	
21 to 24 h	6 (2.6%)	2 (2.6%)	8 (4.8%)	3 (4.5%)	0 (0.0%)	2 (3.6%)	0 (0.0%)	21 (3.4%)	
After more than 1 day	7 (3.0%)	6 (7.8%)	8 (4.8%)	1 (1.5%)	0 (0.0%)	1 (1.8%)	0 (0.0%)	23 (3.8%)	
**Duration of symptoms**									0.046
Less than one day	39 (17.0%)	11 (14.3%)	19 (11.3%)	13 (19.7%)	1 (16.7%)	7 (12.7%)	2 (28.6%)	92 (15.1%)	
1 to 3 days	60 (26.1%)	13 (16.9%)	97 (57.7%)	32 (48.5%)	4 (66.7%)	17 (30.9%)	3 (42.9%)	226 (37.1%)	
4 to 7 days	8 (3.5%)	5 (6.5%)	15 (8.9%)	9 (13.6%)	0 (0.0%)	4 (7.3%)	0 (0.0%)	41 (6.7%)	
More than 7 days	5 (2.2%)	4 (5.2%)	6 (3.6%)	2 (3.0%)	0 (0.0%)	3 (5.5%)	0 (0.0%)	20 (3.3%)	
**Response to relieve symptoms**									<0.001
Rest at home	64 (27.8%)	16 (20.8%)	24 (14.3%)	13 (19.7%)	1 (16.7%)	8 (14.5%)	2 (28.6%)	128 (21.0%)	
Take painkillers & rest at home	45 (19.6%)	16 (20.8%)	112 (66.7%)	42 (63.6%)	4 (66.7%)	22 (40.0%)	3 (42.9%)	244 (40.1%)	
Visit clinic without hospitalization	3 (1.3%)	1 (1.3%)	1 (0.6%)	0 (0.0%)	0 (0.0%)	1 (1.8%)	0 (0.0%)	6 (1.0%)	
Hospitalization	0 (0.0%)	0 (0.0%)	0 (0.0%)	1 (1.5%)	0 (0.0%)	0 (0.0%)	0 (0.0%)	1 (0.2%)	

Chi-squared test was used with a *p-*value ˂ 0.05.

**Table 3:** COVID-19 vaccine side effects reported by the participants after the first dose of different vaccine types (*n* = 609).

Side Effects after 1st Dose	Inactivated Vaccine		Viral Vector Vaccine			mRNA Vaccine		Total (*n* = 609) N, %	*p-*Value
	Sinovac (*n* = 230) N, %	Sinopharm (*n* = 77) N, %	AstraZeneca/Oxford (*n* = 168) N, %	Johnson & Johnson (*n* = 66) N, %	Sputnik V (*n* = 6) N, %	Pfizer-BioNTech (*n* = 55) N, %	Moderna (*n* = 7) N, %		
A nose bleed	2 (0.9%)	0 (0.0%)	5 (3.0%)	2 (3.0%)	0 (0.0%)	0 (0.0%)	0 (0.0%)	9 (1.5%)	0.375
A sore or dry throat	8 (3.5%)	4 (5.2%)	21 (12.5%)	11 (16.7%)	0 (0.0%)	6 (10.9%)	0 (0.0%)	50 (8.2%)	0.003
Abdominal pain	11 (4.8%)	5 (6.5%)	20 (11.9%)	7 (10.6%)	0 (0.0%)	4 (7.3%)	0 (0.0%)	47 (7.7%)	0.176
Bleeding gum	0 (0.0%)	1 (1.3%)	0 (0.0%)	0 (0.0%)	0 (0.0%)	0 (0.0%)	0 (0.0%)	1 (0.2%)	0.328
Body sweats for no reason	4 (1.7%)	0 (0.0%)	22 (13.1%)	10 (15.2%)	0 (0.0%)	1 (1.8%)	1 (14.3%)	38 (6.2%)	<0.001
Bruises on the body	7 (3.0%)	3 (3.9%)	13 (7.7%)	4 (6.1%)	0 (0.0%)	1 (1.8%)	0 (0.0%)	28 (4.6%)	0.316
Chest pain	9 (3.9%)	2 (2.6%)	17 (10.1%)	7 (10.6%)	0 (0.0%)	2 (3.6%)	0 (0.0%)	37 (6.1%)	0.059
Chills and shiver	10 (4.3%)	5 (6.5%)	59 (35.1%)	17 (25.8%)	1 (16.7%)	4 (7.3%)	1 (14.3%)	97 (15.9%)	<0.001
Clogged nose	7 (3.0%)	3 (3.9%)	17 (10.1%)	9 (13.6%)	1 (16.7%)	3 (5.5%)	0 (0.0%)	40 (6.6%)	0.014
Cold, numbness and tingling in limbs	12 (5.2%)	6 (7.8%)	29 (17.3%)	17 (25.8%)	2 (33.3%)	4 (7.3%)	0 (0.0%)	70 (11.5%)	<0.001
Cough	10 (4.3%)	4 (5.2%)	23 (13.7%)	12 (18.2%)	2 (33.3%)	7 (12.7%)	0 (0.0%)	58 (9.5%)	0.001
Decreased sleep quality	10 (4.3%)	4 (5.2%)	31 (18.5%)	7 (10.6%)	0 (0.0%)	3 (5.5%)	1 (14.3%)	56 (9.2%)	<0.001
Diarrhea	4 (1.7%)	8 (10.4%)	12 (7.1%)	7 (10.6%)	1 (16.7%)	0 (0.0%)	1 (14.3%)	33 (5.4%)	0.003
Faster or irregular heartbeats	8 (3.5%)	3 (3.9%)	19 (11.3%)	7 (10.6%)	0 (0.0%)	1 (1.8%)	0 (0.0%)	38 (6.2%)	0.014
Runny nose	6 (2.6%)	4 (5.2%)	9 (5.4%)	12 (18.2%)	1 (16.7%)	4 (7.3%)	0 (0.0%)	36 (5.9%)	<0.001
Dizziness	21 (9.1%)	8 (10.4%)	43 (25.6%)	26 (39.4%)	3 (50.0%)	3 (5.5%)	2 (28.6%)	106 (17.4%)	<0.001
Dyspnea	1 (0.4%)	1 (1.3%)	6 (3.6%)	0 (0.0%)	0 (0.0%)	1 (1.8%)	0 (0.0%)	9 (1.5%)	0.237
Fever	17 (7.4%)	9 (11.7%)	80 (47.6%)	35 (53.0%)	4 (66.7%)	9 (16.4%)	2 (28.6%)	156 (25.6%)	<0.001
Haziness or lack-of-clarity in eyesight	6 (2.6%)	0 (0.0%)	19 (11.3%)	6 (9.1%)	0 (0.0%)	1 (1.8%)	0 (0.0%)	32 (5.3%)	0.001
Headache	55 (23.9%)	21 (27.3%)	87 (51.8%)	37 (56.1%)	4 (66.7%)	13 (23.6%)	4 (57.1%)	221 (36.3%)	<0.001
Increase or decrease in blood pressure	3 (1.3%)	3 (3.9%)	4 (2.4%)	5 (7.6%)	0 (0.0%)	1 (1.8%)	0 (0.0%)	16 (2.6%)	0.179
Irritation and allergic skin reactions	4 (1.7%)	4 (5.2%)	5 (3.0%)	1 (1.5%)	0 (0.0%)	1 (1.8%)	0 (0.0%)	15 (2.5%)	0.711
Joints pain	13 (5.7%)	5 (6.5%)	44 (26.2%)	17 (25.8%)	0 (0.0%)	4 (7.3%)	1 (14.3%)	84 (13.8%)	<0.001
Muscle pain (myalgia)	25 (10.9%)	4 (5.2%)	63 (37.5%)	25 (37.9%)	3 (50.0%)	10 (18.2%)	2 (28.6%)	132 (21.7%)	<0.001
Nausea	8 (3.5%)	1 (1.3%)	25 (14.9%)	7 (10.6%)	1 (16.7%)	2 (3.6%)	0 (0.0%)	44 (7.2%)	<0.001
Over sleepiness or laziness	34 (14.8%)	7 (9.1%)	55 (32.7%)	21 (31.8%)	2 (33.3%)	10 (18.2%)	1 (14.3%)	130 (21.3%)	<0.001
Pain or swelling at the injection site	27 (11.7%)	10 (13.0%)	58 (34.5%)	24 (36.4%)	2 (33.3%)	12 (21.8%)	4 (57.1%)	137 (22.5%)	<0.001
Swollen ankles and feet	2 (0.9%)	0 (0.0%)	5 (3.0%)	2 (3.0%)	0 (0.0%)	0 (0.0%)	0 (0.0%)	9 (1.5%)	0.375
Tiredness and fatigue	45 (19.6%)	10 (13.0%)	70 (41.7%)	23 (34.8%)	3 (50.0%)	10 (18.2%)	3 (42.9%)	164 (26.9%)	<0.001
Vomiting	2 (0.9%)	2 (2.6%)	7 (4.2%)	2 (3.0%)	0 (0.0%)	0 (0.0%)	0 (0.0%)	13 (2.1%)	0.33
For Females, menstrual disturbance	11 (4.8%)	6 (7.8%)	14 (8.3%)	7 (10.6%)	0 (0.0%)	2 (3.6%)	2 (28.6%)	42 (6.9%)	0.119
For Males, impotence	0 (0.0%)	0 (0.0%)	0 (0.0%)	1 (1.5%)	0 (0.0%)	0 (0.0%)	0 (0.0%)	1 (0.2%)	0.221

Chi-squared test was used with a *p-*value < 0.05.

**Table 4:** COVID-19 vaccine side effects reported by the participants after the second dose of different vaccine types (*n* = 609).

Side Effects after 2nd Dose	Inactivated Vaccine		Viral Vector Vaccine			mRNA Vaccine		Total (*n* = 609) N, %	*p-*Value
	Sinovac (*n* = 230) N, %	Sinopharm (*n* = 77) N, %	AstraZeneca/Oxford (*n* = 168) N, %	Johnson & Johnson (*n* = 66) N, %	Sputnik V (*n* = 6) N, %	Pfizer-BioNTech (*n* = 55) N, %	Moderna (*n* = 7) N, %		
A nose bleed	0 (0.0%)	1 (1.3%)	2 (1.2%)	0 (0.0%)	0 (0.0%)	0 (0.0%)	0 (0.0%)	3 (0.5%)	0.61
A sore or dry throat	7 (3.0%)	1 (1.3%)	4 (2.4%)	0 (0.0%)	0 (0.0%)	3 (5.5%)	0 (0.0%)	15 (2.5%)	0.569
Abdominal pain	6 (2.6%)	2 (2.6%)	3 (1.8%)	0 (0.0%)	0 (0.0%)	1 (1.8%)	0 (0.0%)	12 (2.0%)	0.894
Bleeding gum	0 (0.0%)	0 (0.0%)	1 (0.6%)	0 (0.0%)	0 (0.0%)	0 (0.0%)	0 (0.0%)	1 (0.2%)	0.854
Body sweats for no reason	5 (2.2%)	0 (0.0%)	5 (3.0%)	1 (1.5%)	0 (0.0%)	1 (1.8%)	1 (14.3%)	13 (2.1%)	0.279
Bruises on your body	2 (0.9%)	0 (0.0%)	2 (1.2%)	0 (0.0%)	0 (0.0%)	1 (1.8%)	0 (0.0%)	5 (0.8%)	0.895
Chest pain	6 (2.6%)	1 (1.3%)	4 (2.4%)	1 (1.5%)	0 (0.0%)	3 (5.5%)	0 (0.0%)	15 (2.5%)	0.798
Chills and shiver	8 (3.5%)	1 (1.3%)	7 (4.2%)	0 (0.0%)	0 (0.0%)	2 (3.6%)	1 (14.3%)	19 (3.1%)	0.339
Clogged nose	5 (2.2%)	1 (1.3%)	2 (1.2%)	0 (0.0%)	0 (0.0%)	3 (5.5%)	0 (0.0%)	11 (1.8%)	0.398
Cold, numbness and tingling in limbs	9 (3.9%)	1 (1.3%)	1 (0.6%)	1 (1.5%)	0 (0.0%)	2 (3.6%)	0 (0.0%)	14 (2.3%)	0.411
Cough	8 (3.5%)	2 (2.6%)	6 (3.6%)	0 (0.0%)	1 (16.7%)	3 (5.5%)	0 (0.0%)	20 (3.3%)	0.334
Decreased sleep quality	6 (2.6%)	1 (1.3%)	7 (4.2%)	0 (0.0%)	0 (0.0%)	3 (5.5%)	1 (14.3%)	18 (3.0%)	0.223
Diarrhea	1 (0.4%)	2 (2.6%)	0 (0.0%)	1 (1.5%)	0 (0.0%)	0 (0.0%)	1 (14.3%)	5 (0.8%)	0.002
Faster or irregular heartbeats	4 (1.7%)	2 (2.6%)	2 (1.2%)	1 (1.5%)	0 (0.0%)	1 (1.8%)	0 (0.0%)	10 (1.6%)	0.989
Runny nose	7 (3.0%)	2 (2.6%)	3 (1.8%)	1 (1.5%)	0 (0.0%)	3 (5.5%)	0 (0.0%)	16 (2.6%)	0.808
Dizziness	14 (6.1%)	0 (0.0%)	15 (8.9%)	4 (6.1%)	2 (33.3%)	5 (9.1%)	1 (14.3%)	41 (6.7%)	0.021
Dyspnea	0 (0.0%)	0 (0.0%)	2 (1.2%)	0 (0.0%)	0 (0.0%)	1 (1.8%)	0 (0.0%)	3 (0.5%)	0.475
Fever	11 (4.8%)	3 (3.9%)	14 (8.3%)	2 (3.0%)	0 (0.0%)	9 (16.4%)	2 (28.6%)	41 (6.7%)	0.005
Haziness or lack-of-clarity in eyesight	6 (2.6%)	0 (0.0%)	5 (3.0%)	0 (0.0%)	0 (0.0%)	1 (1.8%)	1 (14.3%)	13 (2.1%)	0.171
Headache	27 (11.7%)	5 (6.5%)	39 (23.2%)	3 (4.5%)	1 (16.7%)	11 (20.0%)	3 (42.9%)	89 (14.6%)	<0.001
Increase or decrease in blood pressure	1 (0.4%)	1 (1.3%)	2 (1.2%)	0 (0.0%)	0 (0.0%)	1 (1.8%)	0 (0.0%)	5 (0.8%)	0.896
Irritation and allergic skin reactions	2 (0.9%)	1 (1.3%)	2 (1.2%)	0 (0.0%)	0 (0.0%)	0 (0.0%)	0 (0.0%)	5 (0.8%)	0.952
Joints pain	8 (3.5%)	0 (0.0%)	8 (4.8%)	1 (1.5%)	1 (16.7%)	2 (3.6%)	1 (14.3%)	21 (3.4%)	0.125
Muscle pain (myalgia)	13 (5.7%)	4 (5.2%)	22 (13.1%)	1 (1.5%)	2 (33.3%)	10 (18.2%)	2 (28.6%)	54 (8.9%)	<0.001
Nausea	3 (1.3%)	0 (0.0%)	6 (3.6%)	2 (3.0%)	0 (0.0%)	2 (3.6%)	0 (0.0%)	13 (2.1%)	0.515
Over sleepiness or laziness	22 (9.6%)	6 (7.8%)	16 (9.5%)	2 (3.0%)	1 (16.7%)	8 (14.5%)	3 (42.9%)	58 (9.5%)	0.025
Pain or swelling at the injection site	22 (9.6%)	5 (6.5%)	23 (13.7%)	1 (1.5%)	1 (16.7%)	11 (20.0%)	4 (57.1%)	67 (11.0%)	<0.001
Swollen ankles and feet	0 (0.0%)	1 (1.3%)	1 (0.6%)	0 (0.0%)	0 (0.0%)	0 (0.0%)	0 (0.0%)	2 (0.3%)	0.706
Tiredness and fatigue	26 (11.3%)	5 (6.5%)	21 (12.5%)	1 (1.5%)	0 (0.0%)	10 (18.2%)	2 (28.6%)	65 (10.7%)	0.027
Vomiting	2 (0.9%)	0 (0.0%)	2 (1.2%)	1 (1.5%)	0 (0.0%)	0 (0.0%)	1 (14.3%)	6 (1.0%)	0.026
Thrombosis (blood clots)	0 (0.0%)	0 (0.0%)	1 (0.6%)	0 (0.0%)	0 (0.0%)	0 (0.0%)	0 (0.0%)	1 (0.2%)	0.854
For Females, menstrual disturbance	11 (4.8%)	5 (6.5%)	6 (3.6%)	1 (1.5%)	1 (16.7%)	3 (5.5%)	2 (28.6%)	29 (4.8%)	0.039

Chi-squared test was used with a *p-*value < 0.05.

**Table 5:** Factors associated with post-vaccination symptoms and COVID-19 infection among the participants (*n* = 609).

	Post-Vaccination Symptoms				Post-Vaccination COVID-19 Infection			
	Yes (*n* = 397) N, %	NO (*n* = 230) N, %	Total (*n* = 609) N, %	*p-*Value	Yes (*n* = 77) N, %	NO (*n* = 532) N, %	Total (*n* = 609) N, %	*p-*Value
**Gender**				<0.001				0.599
Female	276 (72.8%)	119 (51.7%)	395 (64.9%)		52 (67.5%)	343 (64.5%)	395 (64.9%)	
Male	103 (27.2%)	111 (48.3%)	214 (35.1%)		25 (32.5%)	189 (35.5%)	214 (35.1%)	
**Age**				0.053				0.425
Under 18	10 (2.6%)	4 (1.7%)	14 (2.3%)		0 (0.0%)	14 (2.6%)	14 (2.3%)	
18–29	265 (69.9%)	182 (79.1%)	447 (73.4%)		58 (75.3%)	389 (73.1%)	447 (73.4%)	
30–39	52 (13.7%)	22 (9.6%)	74 (12.2%)		9 (11.7%)	65 (12.2%)	74 (12.2%)	
40–49	37 (9.8%)	10 (4.3%)	47 (7.7%)		9 (11.7%)	38 (7.1%)	47 (7.7%)	
50–59	11 (2.9%)	8 (3.5%)	19 (3.1%)		1 (1.3%)	18 (3.4%)	19 (3.1%)	
60–69	3 (0.8%)	1 (0.4%)	4 (0.7%)		0 (0.0%)	4 (0.8%)	4 (0.7%)	
More than 69	1 (0.3%)	3 (1.3%)	4 (0.7%)		0 (0.0%)	4 (0.8%)	4 (0.7%)	
**Comorbidities**				0.209				0.372
Yes	178 (47.0%)	96 (41.7%)	274 (45.0%)		31 (40.3%)	243 (45.7%)	274 (45.0%)	
No	201 (53.0%)	134 (58.3%)	335 (55.0%)		46 (59.7%)	289 (54.3%)	335 (55.0%)	
**Medications**				0.66				0.659
Yes	73 (19.3%)	41 (17.8%)	114 (18.7%)		13 (16.9%)	101 (19.0%)	114 (18.7%)	
No	306 (80.7%)	189 (82.2%)	495 (81.3%)		64 (83.1%)	431 (81.0%)	495 (81.3%)	
**Smoker**				0.054				0.48
Yes	59 (15.6%)	50 (21.7%)	109 (17.9%)		16 (20.8%)	93 (17.5%)	109 (17.9%)	
No	320 (84.4%)	180 (78.3%)	500 (82.1%)		61 (79.2%)	439 (82.5%)	500 (82.1%)	
**Received flu shot**				0.707				0.327
Yes	84 (22.2%)	48 (20.9%)	132 (21.7%)		20 (26.0%)	112 (21.1%)	132 (21.7%)	
No	295 (77.8%)	182 (79.1%)	477 (78.3%)		57 (74.0%)	420 (78.9%)	477 (78.3%)	
**Previous Allergies**				0.187				0.816
Yes	49 (12.9%)	19 (8.3%)	68 (11.2%)		9 (11.7%)	59 (11.1%)	68 (11.2%)	
No	330 (87.1%)	211 (91.7%)	541 (88.8%)		68 (88.3%)	473 (88.9%)	541 (88.8%)	
**Previous COVID-19 infection**				0.232				0.005
Yes	140 (36.9%)	74 (32.2%)	214 (35.1%)		38 (49.4%)	176 (33.1%)	214 (35.1%)	
No	239 (63.1%)	156 (67.8%)	395 (64.9%)		39 (50.6%)	356 (66.9%)	395 (64.9%)	
**No. of vaccine doses**				0.011				0.066
Single dose	72 (19.0%)	24 (10.4%)	96 (15.8%)		18 (23.4%)	78 (14.7%)	96 (15.8%)	
Two doses	301 (79.4%)	199 (86.5%)	500 (82.1%)		59 (76.6%)	441 (82.9%)	500 (82.1%)	
More than two doses	6 (1.6%)	7 (3.0%)	13 (2.1%)		0 (0.0%)	13 (2.4%)	13 (2.1%)	
**Type of vaccine**				<0.001				0.414
Sinovac	112 (29.6%)	118 (51.3%)	230 (37.8%)		29 (37.7%)	201 (37.8%)	230 (37.8%)	
Sinopharm	33 (8.7%)	44 (19.1%)	77 (12.6%)		10 (13.0%)	67 (12.6%)	77 (12.6%)	
AstraZeneca/Oxford	137 (36.1%)	31 (13.5%)	168 (27.6%)		19 (24.7%)	149 (28.0%)	168 (27.6%)	
Johnson & Johnson	56 (14.8%)	10 (4.3%)	66 (10.8%)		12 (15.6%)	54 (10.2%)	66 (10.8%)	
Sputnik V	5 (1.3%)	1 (0.4%)	6 (1.0%)		2 (2.6%)	4 (0.8%)	6 (1.0%)	
Pfizer-BioNTech	31 (8.2%)	24 (10.4%)	55 (9.0%)		5 (6.5%)	50 (9.4%)	55 (9.0%)	
Moderna	5 (1.3%)	2 (0.9%)	7 (1.1%)		0 (0.0%)	7 (1.3%)	7 (1.1%)	

Chi-squared test was used with a *p-*value < 0.05.

**Table 6:** Odds Ratio for the occurrence of post-vaccination symptoms.

Variable	OR	95% C.I.		*p-*Value
		Lower	Upper	
Gastrointestinal Disorders	4.545	1.274	16.218	<0.05
Gender (Female)	0.346	0.237	0.506	<0.001
Type of vaccine:				<0.001
Pfizer	1.239	0.668	2.296	0.497
Sinopharm	0.703	0.408	1.209	0.202
AstraZeneca	5.144	3.159	8.376	<0.001
Johnson & Johnson	5.965	2.852	12.473	<0.001
Moderna	2.632	0.478	14.493	0.266
Sputnik	5.763	0.633	52.427	0.12

OR = odds ratio, C.I.: confidence interval, *p-*value < 0.05.

**Table 7:** Mental health symptoms and association with COVID-19 vaccination receipt among participants (*n* = 609).

Mental Health		AstraZeneca/Oxford (*n* = 168) N, %	Pfizer-BioNTech (*n* = 55) N, %	Sinopharm (*n* = 77) N, %	Sinovac (*n* = 230) N, %	Johnson & Johnson (*n* = 66) N, %	Moderna (*n* = 7) N, %	Sputnik V (*n* = 6) N, %	Total (*n* = 609) N, %	*p-*Value
**Feeling nervous, or anxious**	Not at all	130 (21.3)	35 (5.7)	53 (8.6)	158 (26)	50 (8.2)	6 (0.9)	5 (0.8)	437 (71.7)	0.759
	Several days	24 (3.9)	14 (2.2)	17 (2.7)	43 (7)	8 (1.3)	1 (0.1)	0	107 (17.5)	
	More than half the days	6 (0.9)	3 (0.4)	3 (0.4)	14 (2.2)	3 (0.4)	0	1 (0.1)	30 (4.9)	
	Nearly every day	8 (1.3)	3 (0.4)	4 (0.6)	15 (2.4)	5 (0.8)	0	0	35 (5.7)	
**Controlling worrying**	Not at all	132 (21.6)	38 (6.2)	52 (8.5)	159 (26.1)	55 (9)	6 (0.9)	4 (0.6)	446 (73.2)	0.279
	Several days	20 (3.2)	14 (2.2)	18 (3)	44 (7.2)	6 (0.9)	1 (0.1)	1 (0.1)	104 (17.1)	
	More than half the days	6 (0.9)	2 (0.3)	3 (0.4)	13 (2.1)	0	0	0	24 (3.9)	
	Nearly every day	10 (1.6)	1 (0.1)	4 (0.6)	14 (2.2)	5 (0.8)	0	1 (0.1)	35 (5.7)	
**Feeling down, depressed or hopeless**	Not at all	114 (18.6)	35 (5.7)	49 (8)	133 (22)	46 (7.5)	5 (0.8)	5 (0.8)	387 (63.5)	0.766
	Several days	31 (5.1)	9 (1.4)	17 (2.7)	58 (9.5)	12 (1.9)	2 (0.3)	0	129 (21.1)	
	More than half the days	13 (2.1)	6 (0.9)	6 (0.9)	16 (2.6)	3 (0.4)	0	0	44 (7.2)	
	Nearly every day	10 (1.6)	5 (0.8)	5 (0.8)	23 (3.7)	5 (0.8)	0	1 (0.1)	49 (8)	
**Little interest or pleasure**	Not at all	117 (19.1)	32 (5.2)	47 (7.7)	130 (21.3)	44 (7.2)	5 (0.8)	3 (0.4)	378 (62.1)	0.521
	Several days	29 (4.7)	12 (1.9)	21 (3.4)	54 (8.8)	12 (1.9)	1 (0.1)	2 (0.3)	131 (21.5)	
	More than half the days	11 (1.8)	3 (0.4)	5 (0.8)	19 (3.1)	3 (0.4)	1 (0.1)	1 (0.1)	43 (7.1)	
	Nearly every day	11 (1.8)	8 (1.3)	4 (0.6)	27 (4.4)	7 (1.1)	0	0	57 (9.3)	

Chi-squared test was used with a *p-*value < 0.05.

## Data Availability

Not applicable.
